# Radioprotective effects of asiaticoside and asiatic acid in neural stem cells derived from human stem cells from apical papilla through increasing dose-reduction factor and their lowering effects on SH-SY5Y cell viability

**DOI:** 10.1371/journal.pone.0325480

**Published:** 2025-06-04

**Authors:** Taweesak Tangrodchanapong, Apisada Jiso, Pimolpun Changkaew, Suphalak Khachonkham, Amarin Thongsuk, Tarinee Chodchavanchai, Phisit Khemawoot, Nisarat Ruangsawasdi, Permphan Dharmasaroja, Anupong Thongklam Songsaad

**Affiliations:** 1 School of Radiological Technology, Faculty of Health Science Technology, Chulabhorn Royal Academy, Bangkok, Thailand; 2 Chakri Naruebodindra Medical Institute, Faculty of Medicine Ramathibodi Hospital, Mahidol University, Samut Prakarn, Thailand; 3 Division of Radiation Oncology, Department of Diagnostic and Therapeutic Radiology, Faculty of Medicine Ramathibodi Hospital, Mahidol University, Bangkok, Thailand; 4 Department of Anatomy, Faculty of Science, Mahidol University, Bangkok, Thailand; 5 Department of Pharmacology, Faculty of Dentistry, Mahidol University, Bangkok, Thailand; 6 Department of Anatomy, Faculty of Dentistry, Mahidol University, Bangkok, Thailand; University of Bari: Universita degli Studi di Bari Aldo Moro, ITALY

## Abstract

**Objective:**

To investigate the effects of asiaticoside (AS) and asiatic acid (AA) against radiotherapy on neural stem cells induced from human stem cells from apical papilla (NSCs-hSCAPs) through dose-reduction factor (DRF) evaluation and their radiosensitization on human neuroblastoma SH-SY5Y cells.

**Methods:**

NSCs-hSCAPs were treated with AS or AA (0–500 μM) and radiation (0–8 Gy). Isolated hSCAPs were verified mesenchymal stem cells (MSCs) properties according to standard protocol. Subsequently, NSCs-hSCAPs were characterized by Cresyl violet staining and immunocytochemistry. A culture plate containing the cells was embedded into the solid water and bolus phantom. After CT simulation and treatment planning, dose uniformity to the plate was evaluated. X-ray, AS, and AA toxicity were investigated using cell viability (MTT) assay. Finally, DRF50 was calculated from dose-response curves at 50% cell viability for both cell lines.

**Results:**

hSCAPs presented MSCs markers. NSCs-hSCAPs were successfully generated due to the Nissl substance, Nestin, and SOX2 positively stained. Dose homogeneity was represented as isodose at 100% covered the cells in the phantom, suggesting that they were received according to prescribed doses. MTT results revealed that AA was more toxic than AS in both cells. X-ray reduced significantly in a number of tested cells and more radiosensitivity was observed in SH-SY5Y. However, the reduction affected by 4 Gy was diminished after AA or AS at 2 μM applied to NSCs-hSCAPs. Moreover, a significant increase of DRF50 was found at 2 μM of AA (6.72 ± 2.35) and AS (3.84 ± 1.41) in NSCs-hSCAPs whereas it did not show in SH-SY5Y. Interestingly, 20 μM AA could reduce SH-SY5Y cell viability (mean of the cell viability (%) was 25.22 ± 1.53 compared to 30.22 ± 1.46 in the control group), showing a very large in terms of its effect size (Cohen’s *d* value = 1.37).

**Conclusion:**

AA and AS had a specific radioprotective effect on NSCs-hSCAPs without affecting SH-SY5Y. However, AA might be a better therapeutic agent due to expressing a lethal effect on the irradiated cancer cells.

## Introduction

Based on Global Cancer Observation 2020, brain cancer is a leading cause of human death, ranking 12^th^ among all lethal cancers, and impacts patient’s quality of life with a low survival rate [1]. To combat the disease, radiotherapy is thereby utilized in curing or alleviating the patient’s symptoms. Although radiotherapy is an effective modality to mitigate cancer, irradiation to normal tissues surrounding the lesions is inevitable, producing a late-delayed effect of radiation-induced brain injury (RIBI) that has severe irreversible neurological consequences [2]. Amongst these, cognitive deficits problems with short-term and long-term memory are the most common and serious complications after whole-brain radiotherapy (WBRT) [3]. Mechanistically, radiation stimulated apoptosis of neural stem cells (NSCs) located in the dentate gyrus of the hippocampus that has abilities to self-renewal and produce new neurons [4]. Thus, halting such neurogenesis caused RIBI-induced cognitive decline. Recently, glucocorticoids and edaravone have been used to relieve development of the complications and inhibit neuronal cell death [2]. Unfortunately, such drugs provide several side effects [5] and protect brain cancer cells [6]. For this reason, the discovery of drugs or compounds that can protect NSCs from radiotherapy without affecting brain cancer cells is an urgent need and might be therapeutic strategies for cognitive impairment in patients.

Asiatic acid (AA) and asiaticoside (AS), pentacyclic triterpenoids derived from *Centella asiatica*, contain several pharmacological properties [7]. A preclinical study revealed that pretreatment of AA (30 mg/kg) was able to improve kainic acid-induced cognitive deficits through enhancing synaptic and mitochondrial function in the suppression of hippocampal neuronal damage [8]. Conversely, AA elevated caspase and Bcl-2 levels in promoting apoptosis of glioblastoma cells [9]. These anti-tumor activities were also obtained in the AS treatment [10], on the other hand, it protected normal neurons from lipid peroxidation-induced necrosis [11]. Importantly, a study demonstrated that AA and AS pass across the blood-brain barrier (BBB) with high permeability of 70.61 ± 6.60, and 50.94 ± 10.91 x 10^−6^ cm/s, respectively [12]. They also showed no cytotoxicity to the tight junction of the *in vitro* BBB model with more permeable across BBB than central nervous system drugs, suggesting higher possibilities in neuroprotection [12].

Recently, there are many limitations to directly harvesting NSCs from humans. First, the invasive isolation of human NSCs which are derived from embryonic stem cells is an ethical consideration [13]. Second, the isolation of NSCs located in the adult human brain needs complex approaches, and a low number of isolated cells are received [14]. Hence, alternative stem cell sources that can be triggered to generate neurogenic properties and are easily accessible become experimental benefits. In this study, NSCs were developed from human stem cells from apical papilla (hSCAPs) that can provide a wide range of lineages of cells including neurogenic [15]. This demonstrated a novel establishment of the cellular model. Taken together, this work aimed to investigate the effects of AA and AS on irradiated NSCs induced from hSCAPs (NSCs-hSCAPs) and human neuroblastoma cells (SH-SY5Y) by evaluating the radiation dose-reduction factor.

## Materials and methods

### Materials

Asiatic acid (AA, 546712, purity 97%, Sigma-Aldrich, MI, USA) and asiaticoside (AS, 43191, purity ≥ 98.5%, Supelco®, Sigma-Aldrich).

### Cell culture

#### hSCAPs isolation.

The healthy apical papilla tissue was collected from human-impacted third molars of Thai patients (18−21 years old, n = 3) at the Oral and Maxillofacial Surgery Clinical, Dental Hospital, Faculty of Dentistry, Mahidol University, Bangkok, Thailand. The research protocol and ethical consideration were approved by the ethics review committee for the Human Right Related to Human Experimentation of the Faculty of Dentistry/Faculty of Pharmacy and Faculty of Medicine Ramathibodi Hospital, Mahidol University, Thailand (MU-MOU COE 2023/003.1101, Protocol No. MU-MOU 2022/DT152). The procedure was conducted following the Declaration of Helsinki. Written informed consent was obtained for the experiment with human subjects during the recruitment period (11 January 2023–11 January 2025). The enzymatic digestion method was performed to isolate the hSCAPs [16]. The isolated cells were cultured in a proliferation medium, which consisted of Minimum Essential Media (MEM,11900−016, Gibco, Life Technologies, NY, USA), 10% Fetal Bovine Serum (FBS, A5256701, Gibco, Life Technologies), and 1% Antibiotic-Antimycotic (15240062, Gibco, Life Technologies) at 37^o^C, 5% CO_2_, and 95% humidity incubator, and observed the cell morphology under the inverted microscope.

#### Characterization of hSCAPs.

To demonstrate the hSCAPs profiling, the isolated cells were characterized with the MSCs properties. Firstly, the isolated cells were cultured with the proliferation medium. The property of ectomesenchymal origin was investigated by immunofluorescence staining of Beta-III tubulin and Nestin.

Secondly, the isolated cells were cultured in the proliferation medium for 12 days. Every 2 days, the proliferation medium was changed. Then, the colonies were visualized by Giemsa staining.

To investigate multi-linage differentiation ability, the isolated cells were induced with adipogenic, osteogenic, and neurogenic induction medium, respectively. Briefly, the isolated cells were seeded at the density of 2x10^4^ cells/well into 24-well plates. After they reached 80% confluence, the cells were induced by an adipogenic induction medium for 3 weeks, osteogenic induction for 3 weeks, and neurogenic induction for 30 hours.

The adipogenic induction medium consists of MEM supplemented with 10% FBS, 1% antibiotic-antimycotic, 0.5 mM 3-isobutyl-1-methylxanthine (I5879, Sigma-Aldrich, MO, USA), 50 µM indomethacin (I7378, Sigma-Aldrich), 1 µM dexamethasone (D4902, Sigma-Aldrich), and 1 µg/mL insulin (I6634, Sigma-Aldrich). The induction medium was changed every 2 days. After completing the induction, the adipogenic differentiation was detected in the lipid droplets using Oil Red O staining.

The osteogenic induction medium consists of MEM supplemented with 10% FBS, 1% antibiotic-antimycotic, 10 mM β-glycerophosphate (G9422, Sigma-Aldrich), 0.1 µM dexamethasone, and 50 mg/ml ascorbate-2-phosphate (A8960, Sigma-Aldrich). The induction medium was changed every 2 days. After completing the induction, the osteogenic differentiation was observed in the calcification of the extracellular matrix using Alizarin red staining.

The neurogenic induction medium consisted of 2 phases. First, the cells were cultured in the neuronal induction medium phase I which consisted of Dulbecco’s Modified Eagle Medium: Nutrient Mixture F-12 (DMEM/F-12, 12500−039, Gibco, Life Technologies,) supplemented with 10% FBS, 1% antibiotic-antimycotic, 500 µM β-mercaptoethanol (M3148, Sigma-Aldrich), and 10 ng/mL basic fibroblast growth factor (bFGF,13256029, Gibco, Life Technologies) for 24 hours. Consequently, the cells were incubated with phase II neuronal induction medium that consisted of DMEM/F-12 supplemented with 2% Dimethyl sulfoxide (DMSO, D2650, Sigma-Aldrich), 1% antibiotic-antimycotic, and 100 µM butylated hydroxyanisole (B1253, Sigma-Aldrich) for 6 hours. After completing the induction, the neurogenic differentiation was detected in neuronal cell-like morphology and positively stained by Cresyl violet staining.

Consequently, the isolated cells were analyzed by specific MSCs markers including CD73 (344021, Biolegend, CA, USA), CD90 (328109, Biolegend), and CD105 (323209, Biolegend). The CD34 (343607, Biolegend) was used as the negative control (hematopoietic stem cell marker). The cell surface markers expression was performed by the BD FACS Canto^TM^ Flow cytometer (BD Biosciences, CA, USA) and analyzed by BD FACSDiva^TM^ software (BD Biosciences).

#### Neurosphere induction and characterization of NSCs.

The characterized hSCAPs were induced into NSCs under 3D-neurosphere induction by culturing with DMEM/F-12, 2% B-27 (17504044, Gibco, Life Technologies), 1% Antibiotic-Antimycotic, 20 ng/mL bFGF, and 20 ng/mL epidermal growth factor (EGF, PHG0311L, Gibco, Life Technologies) in ultra-low attachment multiple-well plate (3473, Corning, NY, USA) for 5 days [17]. Half of the medium was changed every other day. The 3D-neurosphere was observed under the inverted microscope. The Nissl substance, which is the typical neuronal hallmark of the neuronal cells was revealed by the Cresyl violet staining. Moreover, the immunophenotyping markers of the NSCs were demonstrated by the expression of Nestin and SOX2 following immunocytochemistry. The neurospheres were digested with Accutase enzyme (00-4555-56, Gibco, Life Technologies) to collect the intraspheroidal cells and further use in the next experiment.

#### Immunocytochemistry.

The samples were fixed with 4% paraformaldehyde (158127, Sigma-Aldrich) for 60 minutes, incubated with 20% cold methanol (107018, EMSURE, MERCK, Darmstadt, Germany) in phosphate-buffered saline (PBS, Na_2_HPO_4_, S5136, KH_2_PO_4_, P5655, NaCl, S5886, and KCl, P5405, Sigma-Aldrich) for 5 minutes, and permeabilized with 0.5% Triton X-100 (108603, Sigma-Aldrich) in PBS overnight at 4^o^C. The non-specific blocking with 15% bovine serum albumin (A7030, Sigma-Aldrich) was performed at 4^o^C for 12 hours. Consequently, the specimens were incubated with primary antibodies to detect Nestin (656802, Biolegend), Beta-III tubulin (801201, Biolegend), and SOX2 (Ab92494, Abcam, Cambridge, UK) overnight at 4^o^C. Then, specimens were conjugated with Alexa plus 488 secondary antibodies (A32723, Invitrogen, NY, USA) and Alexa plus 594 secondary antibodies (A21207, Invitrogen) for 4 hours at room temperature. Nuclei were counter-stained and mounted with Prolong^TM^ Diamond Antifade Mountant with DAPI (P36962, Invitrogen). The immunofluorescent staining was revealed and captured under the confocal microscope platforms STELLARIS 5 (Leica Microsystems, Wetzlar, Germany).

#### Cresyl violet staining.

The samples were fixed with 4% paraformaldehyde for 60 minutes and washed with PBS for 5 minutes 2 times and double distilled water for 1 minute. Consequently, the samples were stained with 0.04% Cresyl violet acetate (J64318.09, Thermo Scientific, MA, USA) for 60 minutes. Then, serial dehydration with 90%, 95%, and 100% ethanol (1.00983, EMSURE, MERCK) was performed, respectively.

#### Cultivation of human neuroblastoma cells.

The human neuroblastoma cells (SH-SY5Y, CRL2266, ATCC, VA, USA) were cultured with DMEM/F-12, 10% FBS, and 1% Antibiotic-Antimycotic at 37^o^C, 5% CO_2_, and 95% humidity incubator. The culture medium was changed every 2 days. The sub-culture with trypsin-EDTA (15400054, Gibco) was performed to expand the cells for further experiments.

### Phantom design and treatment planning

**Fig 1:**
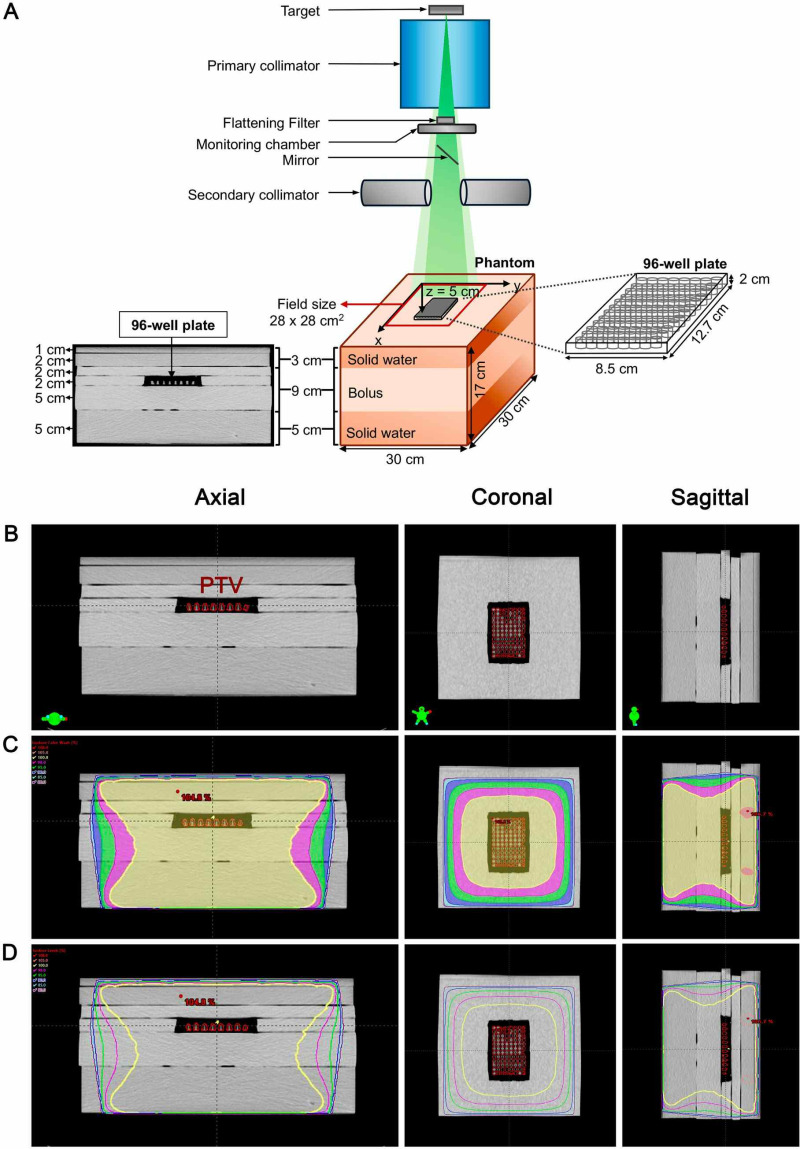
Irradiation set-up of solid water and bolus phantom and treatment planning on CT images. (A) The diagram shows the configuration and irradiation set-up of the phantom. (B – D) Isodose coverages of PTV (96-well plate) on phantom’s CT images by using treatment planning system on axial, coronal, and sagittal plane.

The phantom was designed and constructed for this study to ensure that NSCs-hSCAPs and SH-SY5Y cells received uniform irradiation (Fig 1). The phantom utilized solid water slabs (Gammex 457-CTG, Certified Therapy Grade, WI, USA) and bolus material (Superflab, Nuclear Associates, NY, USA) to configure its structure. In radiotherapy, the solid water is manufactured for irradiating on a linear accelerator (LINAC), using a 6 MV photon, and has depth for ionization for photon, mass-restricted electron stopping power, and linear attenuation coefficient relative to water. These tissue-equivalent properties are also observed in the Superflab bolus which radiation absorption characteristics and density are equivalent to the soft tissue of humans and close to water, respectively [18]. As illustrated in Fig 1A, the size of the phantom was 30 x 30 x 17 cm^3^ (Width (W) x Length (L) x Height (H)). The solid water (5 cm) and the bolus (5 cm) were placed under the 96-well plate as well as the solid water and the bolus with 3 cm and 2 cm thickness, respectively, were placed on top of it. The 96-well plate (8.5 x 12.7 x 1 cm^3^) (W x L x H) (Nunc^TM^, Thermo Scientific) containing the cells in 200 μL culture medium was occupied and had position on the 2 cm-bolus located under the phantom’s superior surface in length of 5 cm. The phantom was imaged by a CT simulator (GE Healthcare Optima CT580 W16 Slice Computed Tomography System, IL, USA). Subsequently, obtained CT images were delivered to Eclipse^TM^ Treatment Planning Software, version 16.1 (Varian Medical System, CA, USA). The 96-well plate containing 200 μL culture medium was contoured as planning target volume (PTV) on all CT slices (Fig 1B). The PTV received different radiation doses as shown in the four plans, with irradiation doses ranging from 0 to 8 Gy, corresponding to different monitor units. Table 1 represents beam data and MUs in the plans. In all designed treatment plans, anterior-posterior (AP) and posterior-anterior (PA) beams with a field size of 28 × 28 cm² (W × L) and a source-to-axis distance of 100 cm are utilized to ensure a homogeneous dose distribution and comprehensive coverage of the PTV. Their field weights were different to adjust dose homogeneity on the PTV.

**Table 1:** Treatment planning information and set-up of the phantom.

Conditions	Beam data	AP beam	PA beam
Common conditions	Machine	TrueBeam	TrueBeam
	Energy	6 MV	6 MV
	Dose Rate	300 MU/min	300 MU/min
	Blocks/MLC	No/No	No/No
	Wedge name	Open field	Open field
	Cough (Lat, Vert, Long (cm)	0.00,9.45,127.28	0.00,9.45,127.28
	Isocenter (L-R, I-S, A-P) (cm)	0.00,0.00,0.00	0.00,0.00,0.00
	Gantry (°)	0.0	180.0
	Collimator (°)	0.0	0.0
	SSD (cm)	94.8	90.4
	Field size (cm)	28.0 x 28.0	28.0 x 28.0
	Collimator size (cm)	X1 = 14.0, X2 = 14.0 Y1 = 14.0, Y2 = 14.0	X1 = 14.0, X2 = 14.0 Y1 = 14.0, Y2 = 14.0
	Field Weight (%)	58.0	42.0
MU/fraction	Plan No. 1 (2 Gy)	116	98
	Plan No. 2 (4 Gy)	231	196
	Plan No. 3 (6 Gy)	347	293
	Plan No. 4 (8 Gy)	462	391

### Protocol of cell irradiation

The cells were irradiated by using a LINAC (TrueBeam, Varian Medical Systems, CA, USA) at the Faculty of Medicine Ramathibodi Hospital, Mahidol University, Bangkok, Thailand. The cells in each condition were exposed to a single dose of 2, 4, 6, and 8 Gy at a dose rate of 300 cGy/min (MU/min) [19]. A control group received no irradiation (0 Gy) and was under the same stress as the other groups.

### Cell viability (MTT) assay

To determine the effect of AS and AA on cell viability of NSCs-hSCAPs and SH-SY5Y cells, a MTT (3-(4,5-dimethylthiazol-2-yl)-2,5-diphenyl tetrazolium bromide) assay was performed. AS and AA were dissolved in DMSO for a stock solution (100 mM) and serial dilution to a working solution by the basal medium (DMEM/F-12 and 1% Antibiotic-Antimycotic). In brief, the cells (30,000 cells/well) were seeded in 96-well plates for 24 hours and treated with AS or AA at various concentrations (0, 1, 2, 10, 20, 50, 100, 250, and 500 µM) for 24 hours. The cells in the control group were incubated with the basal medium whereas those in the vehicle group were treated with the basal medium supplemented with 0.5% DMSO. After removing the medium, the cells were incubated with MTT (0.5 mg/mL, M5655, Sigma-Aldrich) for 2 hours. The formazan crystal was solubilized by DMSO. The absorbance was measured by a microplate reader (BioTek Epoch, Agilent, CA, USA) at 570 nm (soluble formazan) and 690 nm (background). Cell viability (%) was calculated using the equation below. The half maximal inhibitory concentration (IC_50_) was determined from a dose-response curve visualized using nonlinear regression (curve fitting) by GraphPad Prism (GraphPad Software, LLC, MA, USA).


$$Cell\;viability\;(\;\%)\;=\;\frac{Mean\;OD\;of\;treated\;group\;(OD570-OD690)}{Mean\;OD\;of\;control\;group\;(OD570-OD690)}\;x\;100$$

To investigate the effect of radiotherapy on both cells, the MTT method was carried out. In brief, the cells were seeded in 96-well plates (30,000 cells/well) at 24 or 48 hours. Then, the plates were exposed to different radiation doses (0–8 Gy). After incubating for 24 hours, the irradiated cells were treated with MTT reagent followed by incubation for 2 hours. The percentage of cell viability was exhibited by relative value to the non-irradiated cells.

In a further step, the radioprotective effect of AS and AA was evaluated. Hence, the cells were pre-treated with non-toxic concentrations of AS or AA for 24 hours. After removing the compounds, the treated cells were irradiated with distinct exposures (0–8 Gy) by a LINAC and were then incubated for another 24 hours. After that, cell viability was tested using the MTT assay. The viability percent was represented by relative value to the untreated and non-irradiated cells.

### Calculation of dose-reduction factor

To assess the radioprotective capacity of AS and AA, an investigation of the dose-reduction factor (DRF) was required. First, the expressing cell viability (%) for each cell treated or untreated with AA or AS, followed by irradiation, was plotted as a dose-response curve. Therefore, the mean lethal dose (LD50) which is the radiation dose value causing the death of 50% of cells could be determined. Such LD50 was then calculated to DRF50 according to the following equation [20]:


$$DRF50\;=\;\frac{LD50\;drug\;group}{LD50\;control\;group}$$

### Statistical analysis

The data are reported as mean ± standard error from the mean (SEM) of three independent experiments. The number of samples is n = 9 per experimental group.

One-way ANOVA (Tukey’s multiple comparisons) was used for statistical analysis using GraphPad Prism. A *p*-value < 0.05 was considered statistically significant.

## Results

### PTV’s radiation dose uniformity

To ensure that the cells received uniformity of irradiation, the phantom was imaged by a CT simulator, and the contoured PTV (96-well plate) on its CT slices was delivered to the medical treatment planning. As illustrated in Fig 1C and 1D, the area of the PTV (37.6 cm^3^) on the designed plans was covered in 100% isodose in the absence of hot and cold spots (ranging from −5% to +7% of the prescribed dose according to The International Commission on Radiation Units and Measurements (ICRU) Report 50 [21]. Dose statistics reported that the PTV coverage was 99.3% with a mean dose of 101.9%. Therefore, it is likely that the PTV would receive close to 100% of the prescribed radiation doses.

### Characterization of hSCAPs

**Fig 2:**
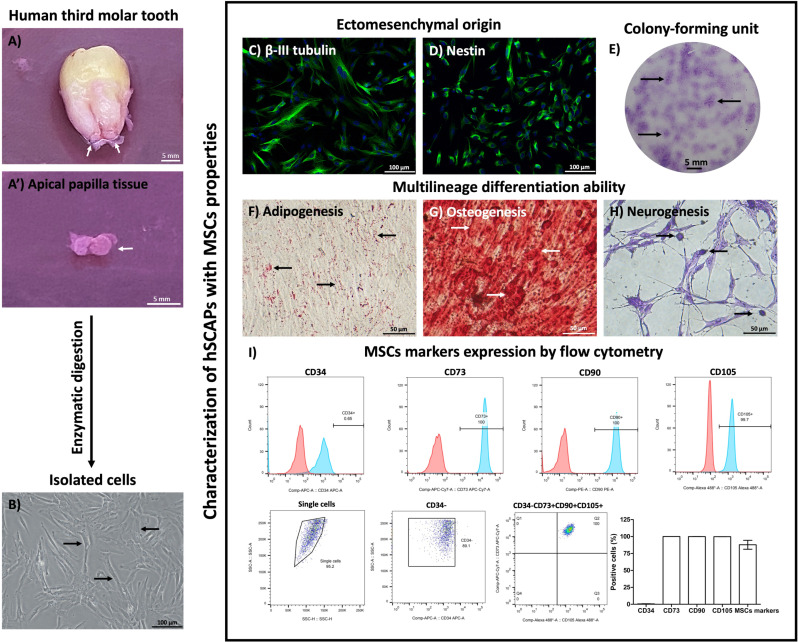
Characterization of hSCAPs. (A) The human third molar teeth presented the apical papilla tissue (white arrows) and (A’) the dissected apical papilla tissue (white arrow). (B) The isolated cells from human apical papilla tissue presented adherent ability and fibroblast-like shape (black arrows). (C – D) The ectomesenchymal origin was revealed by β-III tubulin and Nestin immunofluorescent staining. (E) Colony-forming unit (black arrows) represented their self-renewal ability. (F – H) Multi-linage differentiation abilities were demonstrated by adipogenesis (black arrows indicate lipid droplets), osteogenesis (white arrows indicate extracellular calcium nodules), and neurogenesis (black arrows indicate Nissl bodies), respectively. (I) The cell surface antigen molecules profiling of MSCs was investigated by flow cytometry. Scale bars: A, A’, E = 5 mm, B – D = 100 µm, and F – H = 50 µm.

The human third molar teeth (Fig 2A), which present the apical papilla tissue (Fig 2A’) were collected and performed enzymatic digestion. An analysis was conducted to determine the properties of MSCs. The isolated cells had a fibroblast-like shape and were able to grow on culture vessels to represent plastic adherence ability (Fig 2B). Without administration of neurogenic induction, immunofluorescence staining confirmed that the cells presented ectomesenchymal origin, as they positively stained for β-III tubulin (Fig 2C) and Nestin (Fig 2D). The isolated cells can form colonies with positively stained Giemsa dye, indicating their self-renewal ability (Fig 2E). Additionally, under optimal differentiation-inducing conditions, these isolated cells demonstrated the accumulation of lipid droplets (Fig 2F), the extracellular calcified nodules (Fig 2G), and exhibited Nissl substance (Fig 2H) as revealed through Alizarin red, Oil Red O, and Cresyl violet staining, respectively. These results indicated that the isolated cells demonstrated the multi-lineage differentiation ability. Moreover, flow cytometry profiling of the cell surface antigen molecule of these cells demonstrated highly expressed positive markers for MSCs, including CD73 (100), CD90 (99.97 ± 0.03342), and CD105 (99.8 ± 0.05774) as indicated by the high intensity of histograms. Moreover, these cells that negatively expressed CD34 (0.62 ± 0.1595) and co-expressed CD34-, CD73 + , CD90 + , and CD105 + were a major population (87.86 ± 6.529) (Fig 2I). Taken together, the isolated cells-derived human apical papilla tissue exhibited properties of MSCs, verified as hSCAPs.

### Characterization of NSCs-hSCAPs

**Fig 3:**
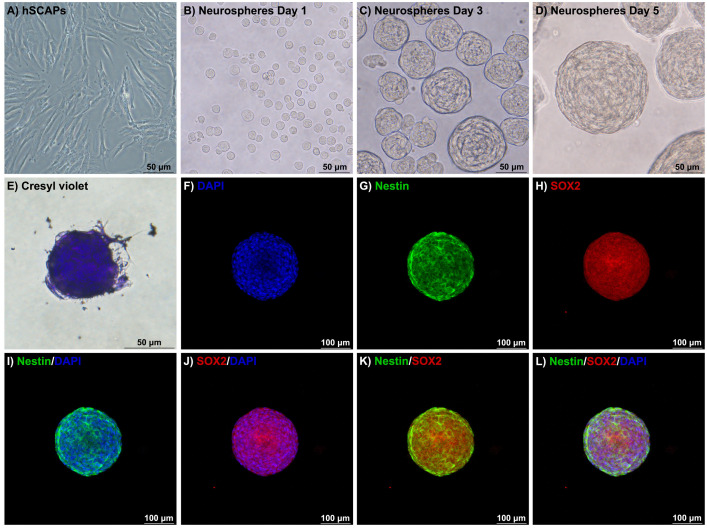
Characterization of NSCs-hSCAPs. The dynamic change of cell morphology of NSCs-hSCAPs. (A) The hSCAPs presented the adherent flattened-shaped cells. (B – D) Under the neural-inducing environment, cells were suspended and aggregated into the 3D-neurosphere, which consisted of intraspheroidal cells. (E) The Cresyl violet staining revealed that these intraspheroidal cells demonstrated the dark purple of the Nissl substance. (F – L) Immunofluorescence profiling of NSCs-hSCAPs under 3D neurosphere induction. **(G – H)** The positive expression of the early neuronal stage, Nestin and SOX2 was observed, respectively. Scale bars: A – E = 50 µm, and F – L = 100 µm.

The characterized hSCAPs were induced into the NSCs under 3D neurosphere formation. These cells changed their cell morphology from the typical fibroblast-like shape (Fig 3A) into the free-floating cells (Fig 3B) under the administration of the NSCs induction medium. Moreover, the free-floating cells were aggregated into the cluster of neuronal cells, which are called “neurospheres”. The size of neurospheres increased in a time-dependent manner on day 3 (Fig 3C) and day 5 (Fig 3D), respectively. The neurospheres consisted of intraspheroidal cells and further characterized their NSCs properties. Firstly, the intraspheroidal cells were positively stained with Cresyl violet dye to reveal the Nissl substance (Fig 3E). The nuclei localization was presented with DAPI staining (Fig 3F). Interestingly, immunofluorescent staining demonstrated that the positive expression of Nestin (Fig 3G) and SOX2 (Fig 3H), which were the NSCs markers was observed in neurospheres. Furthermore, the intraspheroidal cells were co-positively expressed Nestin, SOX2, and DAPI (Fig 3I - 3L). Taken together, these findings suggested that the hSCAPs were differentiated into the NSCs under 3D neurosphere induction.

### Effect of AS and AA on cell viability

**Fig 4:**
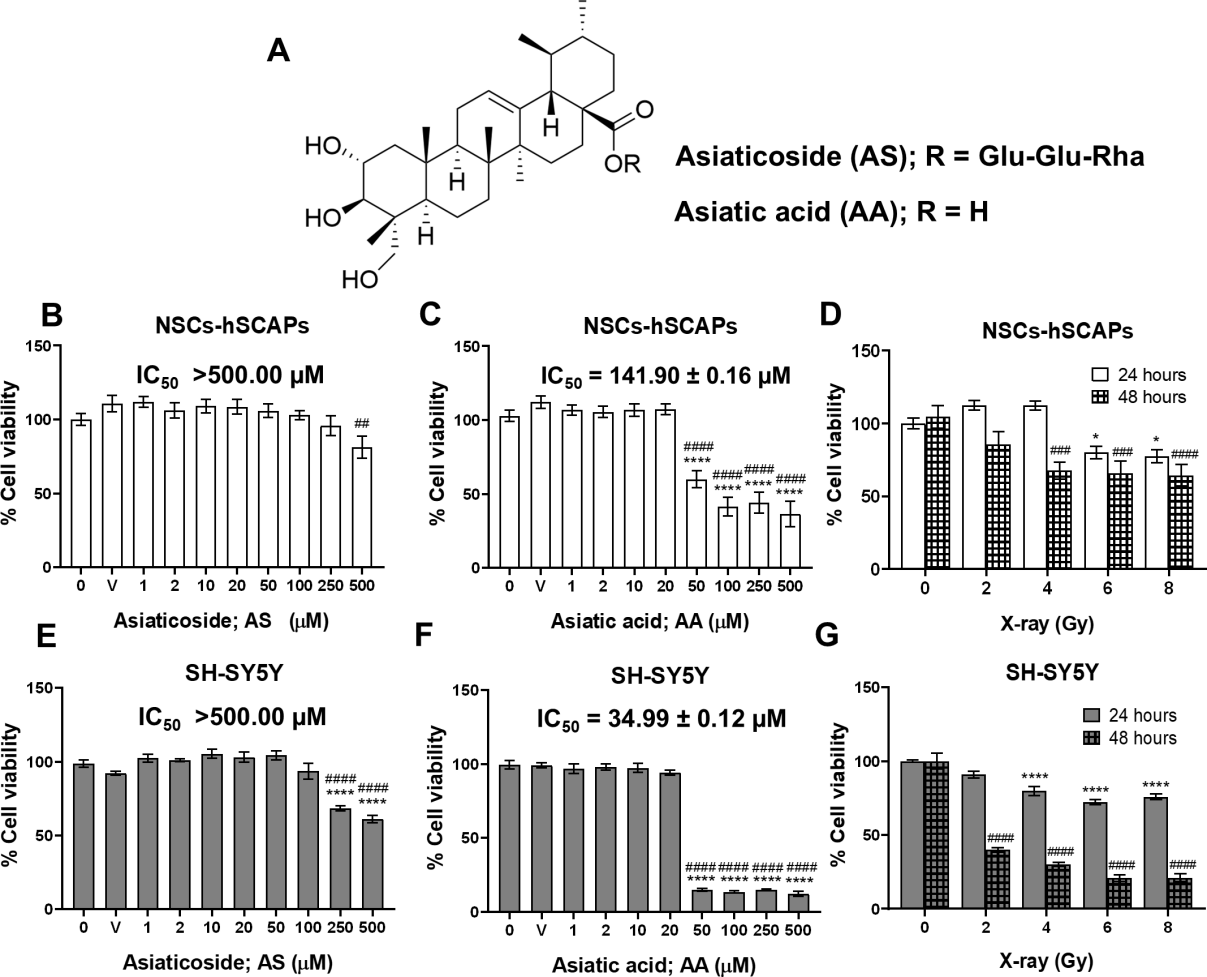
Effect of AS, AA, and X-ray radiation on cell viability (%). (A) The chemical structures of AS and AA. (B – D) Cell viability (%) of NSCs-hSCAPs treated with AS (0-500 µM) at 24 hours, AA (0-500 µM) at 24 hours, and X-ray (0-8 Gy) at 24 and 48 hours, respectively. (E – G) Cell viability (%) of SH-SY5Y treated with AS (0–500 µM) at 24 hours, AA (0–500 µM) at 24 hours, and X-ray (0–8 Gy) at 24 and 48 hours, respectively. Results are represented as mean cell viability (%) ± SEM from three independent experiments. The number of samples is n = 9 for each condition. Error bars represent SEM. **** = *p *< 0.0001 compared to the control group, ^##^ = *p *< 0.01 compared to the vehicle, ^####^ = *p *< 0.0001 compared to the vehicle (Fig 4B, 4C, 4E, and 4F). * = *p *< 0.05, **** = *p* < 0.0001 compared to the control group at 24 hours, ^###^ = *p *< 0.001, ^#### ^= *p *< 0.0001 compared to the control group at 48 hours (Fig 4D and 4G).

The MTT results showed that AA decreased significantly cell viability (%) more than AS in both cells. The structure of AS and AA is illustrated, with AS being the glycoside form of AA (Fig 4A). In NSCs-hSCAPs, AS decreased significantly cell viability (%) at 500 µM with the mean of IC_50_ greater than 500.00 µM (Fig 4B). Conversely, AA reduced cell viability (%) in a dose-dependent manner (*p* < 0.0001) in NSCs-hSCAPs, with the mean of IC_50 _± SEM value of 141.90 ± 0.16 µM (Fig 4C). In SH-SY5Y cells, AS significantly reduced cell viability (%) at 250 and 500 µM (*p* < 0.0001). However, the mean of IC_50_ of AS was greater than 500.00 µM (Fig 4E). On the other hand, AA showed a dramatic reduction in cell viability (%) in SH-SY5Y cells. The %cell viability of AA was less than 50% at concentrations of 50–500 µM (*p* < 0.0001), and the mean of IC_50_ ± SEM value of AA was 34.99 ± 0.12 µM (Fig 4F).

### Effect of radiotherapy on cell viability

To examine the radiation effects on cell viability, both cell lines were exposed to various doses (0–8 Gy) at 24 or 48 hours. The results showed that after NSCs-hSCAPs were irradiated, their cell viability (%) was lower in a radiation dose-dependent manner at 24 and 48 hours. However, a dramatic decrease in the cell viability (%) was observed after irradiation at 48 hours. As illustrated in Fig 4D, radiation doses of 4, 6, and 8 Gy exhibited significant effects on the cell viability (*p* < 0.001 for 4 and 6 Gy as well as *p* < 0.0001 for 8 Gy). Similar results were obtained in irradiated SH-SY5Y cells (Fig 4G). Interestingly, it is likely that SH-SY5Y cells were more sensitive to radiation than NSCs-hSCAPs as lower cell viability (%) was expressed.

### Protective effects of AS and AA against radiotherapy

**Fig 5:**
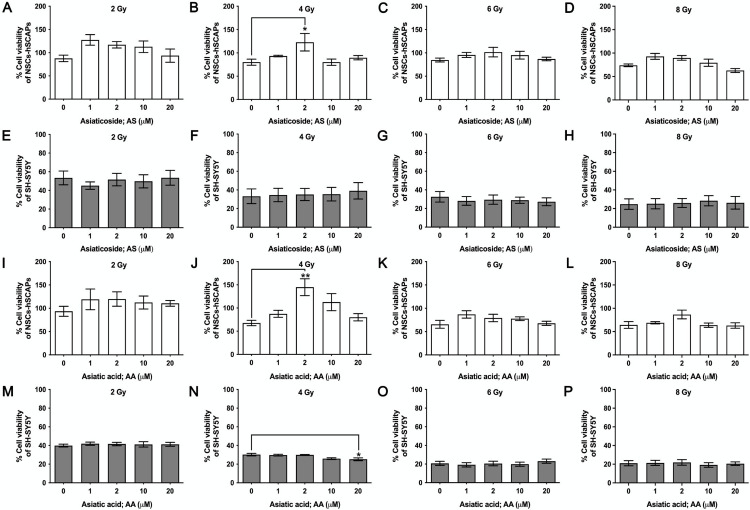
Cell viability (%) of irradiated NSCs-hSCAPs and SH-SY5Y in AS and AA treatment. Effect of AS and AA on X-ray radiation (6 MV)-induced reduction of cell viability (%) in NSCs-hSCAPs and SH-SY5Y cells. Cell viability (%) of the irradiated NSCs-hSCAPs (A – D) or SH-SY5Y cells (E – H) with 2 - 8 Gy in the presence of AS at non-toxic concentrations (0 - 20 µM). Cell viability (%) of the irradiated NSCs-hSCAPs (I – L) or SH-SY5Y cells (M – P) with 2 - 8 Gy in the presence of AA at non-toxic concentrations (0 - 20 µM). The cell viability (%) is represented as mean ± SEM from three independent experiments. The number of samples is n = 9 for each condition. * *p* < 0.05 and ** *p* < 0.01 vs. untreated control NSCs-hSCAPs or SH-SY5Y cells.

Treatment of AS and AA in non-toxic concentrations (1–20 μM) before radiation exposure with various doses (0–8 Gy) on NSCs-hSCAPs was carried out for radioprotective investigation. The MTT results revealed that AS-treated NSCs-hSCAPs did not show a significant increase in cell viability (%) compared to control at radiation doses of 2, 6, and 8 Gy (Fig 5A, 5C, and 5D), respectively. However, AS at 2 μM showed an increase compared to untreated cells at 4 Gy (*p* < 0.05) (Fig 5B). On the other hand, AS at the same concentration did not affect treated SH-SY5Y cells at 4 Gy and other radiation doses as shown in the same level of cell viability (%) to control (Fig 5E - 5H). This suggested a selective effect to protect NSCs-hSCAPs of AS.

In addition to AS, AA was also tested on both cells. Although exposed NSCs-hSCAPs with radiation exhibited a significant decrease in cell viability (%), AA at the non-toxic concentration protected the irradiated cell. As demonstrated in Fig 5J, AA at 2 μM increased significantly cell viability (%) against 4 Gy when compared to untreated cells (*p* < 0.01). In contrast, AA at the same concentration in SH-SY5Y cells did not show the protective effect (Fig 5N). Moreover, there was a significant decrease of cell viability (%) when 20 μM AA was treated (*p* < 0.05). In addition, based on Cohen’s formula [22], the *d* value of AA at 20 μM was 1.37, interpreting that the very large magnitude was observed in terms of its effect size. This indicated a potential effect in protecting normal cells and the anti-tumor effect of AA. Additionally, although at other radiation doses AA at 2–20 μM did not affect cell viability of NSCs-hSCAPs (Fig 5I, 5K, and 5L), it did not enhance SH-SY5Y cell viability in comparison with control (Fig 5M, 5O, and 5P).

### DRF investigation

**Fig 6:**
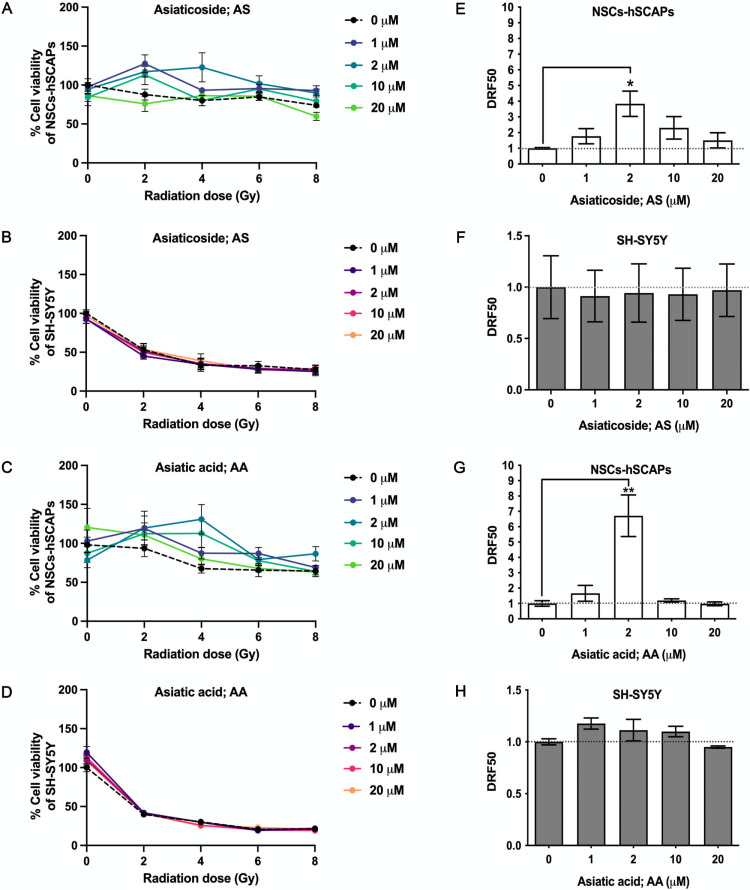
Dose-response curves and DRF50 of NSCs-hSCAPs and SH-SY5Y in the presence of AS and AA. Dose-response curve of NSCs-hSCAPs treated with AS (A) or AA (C) and SH-SY5Y cells treated with AS (B) or AA (D). (A – D) The results showed the mean of cell viability (%) ± SEM at each radiation dose value from three repeated assays. DRF50 was quantified from the dose-response curves. DRF50 of AS in NSCs-hSCAPs (E) and SH-SY5Y cells (F). DRF50 of AA in NSCs-hSCAPs (G) and SH-SY5Y cells (H). (E – H) The results are the mean of DRF50 ± SEM from three independent experiments. The number of samples is n = 9 for each condition. * *p* < 0.05 and ** *p* < 0.01 vs. untreated control NSCs-hSCAPs or SH-SY5Y cells.

DRF50 was calculated from dose-response curves at 50% cell viability of both cells untreated and treated with AS or AA (Fig 6A - 6D). DRF50 is utilized for assessing the radioprotective effect [20]. In AS-treated NSCs-hSCAPs, DRF50 increased in the range of 1–2 μM and reached the highest at 2 μM before slightly decreasing when concentrations were higher (10–20 μM) (Fig 6E). The findings showed that AS enhanced DRF50 with a value greater than one (> 1) for all non-toxic concentrations (1–20 μM), indicating radioprotective capacity. Nevertheless, amongst these concentrations, AS at 2 μM exhibited a significant increment of DRF50 (3.84 ± 1.41) compared to non-treatment (*p* < 0.05). Conversely, AS at the same concentration did not increase the DRF50 value in treated SH-SY5Y (Fig 6F). Additionally, other concentrations of AS did not affect altering DRF50 values compared to the control. This indicated a specific radioprotective effect of AS on NSCs-hSCAPs.

In AA-treated NSCs-hSCAPs, the trend of DRF50 values was similar to the AS-treated cells. The results showed that DRF50 values (>1) were observed in AA in the concentrations of 1, 2, and 10 μM (Fig 6G). Interestingly, there was a significant enhancement of DRF50 (6.72 ± 2.35) when the cell was exposed to 2 μM (*p* < 0.01). Moreover, the enhancing values of DRF50 were not found in the treated SH-SY5Y with AA for all non-toxic concentrations. As illustrated in Fig 6H, there were no significant differences in DRF50 values between the untreated and treated cells. Therefore, AA had specifically radioprotective potential for NSCs-hSCAPs and showed more capacity than AS in the same condition.

## Discussion

The study aimed to investigate the radioprotective effects of AS and AA on NSCs-hSCAPs and the radiosensitizing effect on SH-SY5Y. In this issue, a solid water and bolus phantom was firstly designed and created. After CT images of the phantom were prepared and contoured PTV (96-well plate) was done, it was found that dose homogeneity to the PTV was obtained, suggesting that the cells received according to the prescribed radiation doses. Additionally, applying the bolus, a flexible tissue equivalent material, surrounding the 96-well plate showed a narrow air gap between them, suggesting more suitable than using the polycarbonate plexiglass phantom previously described [20]. Thus, this may minimize an isodose irregularity.

According to adult neurogenesis, the NSCs play important roles in regenerating and replacing damaged neuronal cells. NSCs are the essential progenitors of the central nervous system (CNS) which is differentiated into neurons, astrocytes, and oligodendrocytes [23]. However, the endogenous repairing by these NSCs was limited to only the subventricular zone of the lateral ventricle and the subgranular zone of the dentate gyrus [24]. MSCs of dental origin are the promising source for neuronal regeneration, due to their neuronal differentiation ability [16,25]. In this study, the hSCAPs were collected from the human apical papilla tissue and characterized the MSCs properties provided by The International Society for Cellular Therapy [26]. Importantly, the hSCAPs originated from migratory neural crest stem cells, the progenitor of neuronal cells, which presented a superior and committed neuronal differentiation ability [27]. Moreover, the establishment of hSCAPs was obtained from dental waste with non-invasive protocol, easy accessibility, and low ethical concerns [15].

Recently, various protocols have been developed to differentiate MSCs into neuronal cells [28]. Importantly, the generation of 3D-neurosphere was defined as the potential cellular model for studying CNS diseases [29]. Under 3D-neurosphere-inducing environment provided essential growth factors (EGF and bFGF) which are required for neural induction and further neuronal maturation [30]. In this study, the hSCAPs were induced into the NSCs under 5 days of 3D-neurosphere formation. These intraspheroidal cells were positively expressed in the Nissl body which is the typical neuronal hallmark [31]. Furthermore, identifying biomarkers was necessary to verify the properties of the NSCs [32]. The intraspheroidal cells positively revealed the Nestin and SOX2, representing the early neuronal stage [17]. Therefore, the intraspheroidal cells were characterized as the NSCs.

In the clinic, patients with brain tumors and metastases can be cured by partial-brain radiotherapy or WBRT using conventional fractionation, hypofractionation, or stereotactic radiosurgery (SRS) [33,34]. However, more than 30% of those patients with brain metastasis are struggling with cognitive decline after receiving WBRT [35]. Moreover, each year in the U.S., up to 50% of the patients receiving brain irradiation exhibited cognitive dysfunction. This thereby poses an enormous impact on the patient’s quality of life. Neuroimaging showed that hippocampal atrophy-caused memory deficits are found in patients who receive either fractionated partial-brain irradiation (total dose of 50.4–60 Gy), WBRT, or SRS in curing nasopharyngeal, maxillary, pituitary, and skull base tumors [36,37]. The mechanisms by which X-ray-induced cognitive impairment was mediated through DNA damage, oxidative stress, and neurogenesis inhibition [38]. In the normal brain, NSCs in the hippocampus are responsible for neurogenesis. Therefore, damaged NSCs cause early cognitive decline [39]. The increasing risk of the complication includes high doses, long periods of irradiation, age, and photon treatment [38]. A study using mini-mental state examination found that a high radiation dose (> 30 Gy) for brain irradiation produced more negative effects on cognitive function than a low dose (< 30 Gy) [38]. In comparison between proton and photon therapy, proton scatter outside the boundary of the beam was a faster drop in normal tissues, producing low cognitive deficits [40]. Unfortunately, more than 90% of brain tumors are treated with photons and only 1% of those receive proton treatment [40]. Moreover, only 4.5% of patients receiving WBRT are planned for hippocampal avoidance [41]. Thus, from these problems, the discovery of drugs or compounds that can protect NSCs from high doses of photon therapy is a promising strategy for attenuating cognitive decline in brain tumor patients. AA, a pentacyclic triterpenoid, and its glycoside, AS, are major components of *Centella asiatica*. In this study, AA had moderate cytotoxicity (IC_50_ value 20.00–100.00 µM) against SH-SY5Y cells and low cytotoxicity (IC_50_ = 141.90 µM) in NSCs-hSCAPs [42]. However, the selectivity index (SI) value (IC_50_ non-cancer cell/IC_50_ cancer cell) of AA was 4 and was classified as a strong cytotoxicity (SI ≥ 3) [42,43]. This suggested that AA is a potential compound for developing an antitumor agent with low side effects on normal cells. On the other hand, AS showed low cytotoxicity in both cells.

In our study, 6 MV X-ray photons caused a reduction of cell viability (%) on both cells in a dose-dependent manner. Nevertheless, AS or AA at a non-toxic concentration of 2 μM inhibited the reduction observed in NSCs-hSCAPs exposed to 4 Gy. In parallel, a significant increase of DRF50 value for AS at the same concentration was observed as 3.84 ± 1.41 and that factor was higher up to 6.72 ± 2.35 in 2 μM AA. It thereby indicated the strong protective effect of those compounds against X-ray. Thus, it is possible that AS and AA might lower cognitive decline mostly found in patients receiving high-dose fraction therapy exceeding 2 Gy per fraction [44]. They may be helpful in older patients with low-performance status who are recommended for receiving hypofractionation [45]. Additionally, NSCs in the dentate gyrus with high radiosensitivity might be protected and survive to perform their function in neurogenesis [46] when AS or AA are applied. A study revealed that AS reduced 5J/m_2_ radiation-induced DNA double-strand break in fibroblast and increased total antioxidant capacity level in mice exposed to 5 Gy [47]. In addition to AS, AA treatment has previously shown its neuroprotection by maintaining the anti-oxidant level and mitochondrial activity [48]. These findings might help to explain the plausible mechanisms of AS and AA in protecting NSCs-hSCAPs from X-ray-induced toxicity. However, these possibilities need further experiments. Additionally, in this study, MTT assay was used to measure the cell viability. MTT is known as tetrazolium salts that can quantify the mitochondrial metabolic rate and indirectly reflect the viable cell numbers. Basically, MTT salt is reduced to water-insoluble purple formazan crystal in the metabolically active cells by mitochondrial dehydrogenases, particularly succinate dehydrogenase. For this reason, these salts are commonly utilized for assessing metabolic viability and cell proliferation [49]. As illustrated in Fig 5B and 5J, AS or AA in a concentration of 2 μM had significant effects on increasing cell viability (%) of irradiated NSCs-hSCAPs with values of more than 100%. These effects might be mediated through enhancing the activity of mitochondrial dehydrogenase with unaffected viable cell numbers. However, the increasing proliferative rate of the treated cells is not excluded. Therefore, these plausible mechanisms should be further examined.

It is noteworthy that although AA at the effective concentration of 2 μM showed a strong radioprotective effect on NSCs-hSCAPs, it did not affect SH-SY5Y cell viability at the same condition. It indicated a specific effect on the normal cells. Moreover, AA at 20 μM could significantly reduce the number of SH-SY5Y cells irradiated at 4 Gy, suggesting its antitumor effect. A report demonstrated that AA triggered apoptosis of SH-SY5Y and attenuated glutamate-induced cognitive deficits in mice models [50]. Furthermore, oral administration of AA (10–100 μM) to mice showed a reduction of tumor volume of human glioblastoma cancer and induction of apoptotic pathway [9]. These might explain how AA mediated radiosensitizing effect on SH-SY5Y, but it requires future studies. Although the no affective effect on SH-SY5Y and radioprotective effect on NSCs-hSCAPs were also observed in AS treatment, AA had a higher DRF50 value and enhanced sensitivity to radiation of cancer cells. Thus, AA may be a better candidate for developing a radioprotective agent in improving cognitive dysfunction.

According to Lipinski’s rule of five, the physicochemical properties of biologically active molecules should have no more than 5 hydrogen bond donors (HBD), no more than 10 hydrogen bond acceptors (HBA), molecular mass less than 500 Da, and a partition coefficient (calculated Log P, cLogP) not greater than 5 [51]. The physicochemical data retrieved from SciFinder shows that the AA structure follows Lipinski’s rule. AA has 4 HBD, 5 HBA, a molecular weight of 488.70 Da, and a log P value of 5.75, indicating good permeability. In contrast, AS contains 12 HBD, 19 HBA, a molecular weight of 959.12 Da, and a log P value of 0.089, suggesting low permeability. The lower permeability of AS may result in a less radioprotective effect compared to AA in NSCs-hSCAPs. On the other hand, in exposed SH-SY5Y with 4 Gy, AA exhibited a radiosensitizing effect at a concentration of 20 µM, whereas AS showed no effect. The lack of radiosensitizing effect of AS may be due to the low permeability of AS to the cancer cells.

## Conclusions

AA and AS at a non-toxic concentration of 2 μM are radioprotective agents with high DRF50 values (6.72 ± 2.35 and 3.84 ± 1.41, respectively) in NSCs-hSCAPs. Moreover, the compounds at the same concentration had no affective effect on SH-SY5Y, indicating the selective effect on the normal cells. Additionally, AA at 20 μM showed a significant lethal effect on irradiated SH-SY5Y cells. This suggested a higher capacity of AA for further developing as a therapeutic agent in the treatment of brain tumor patients. In this study, the solid water and bolus phantom was firstly constructed. It could provide dose uniformity to the cells on its CT images; thus, such a designed phantom exhibited a suitable tool for cell irradiation. Furthermore, we firstly accomplished to generate NSCs-hSCAPs as a cellular testing model. Therefore, it will be used as the potential source for other neuroprotective tests.
